# Mental Health Issues Associated With COVID-19 Among the Elderly Population: A Narrative Review

**DOI:** 10.7759/cureus.33081

**Published:** 2022-12-29

**Authors:** Duaa A Bafail

**Affiliations:** 1 Pharmacology Department, King Abdulaziz University, Jeddah, SAU

**Keywords:** digital technologies, social interactions, elderly population, mental health issue, covid-19

## Abstract

Since its discovery in December 2019, coronavirus disease 2019 (COVID-19) has been affecting humanity in economic, social, physical, and psychological manner. During COVID-19, the older population needs special consideration since they have a greater risk of developing serious illnesses. We used a narrative review approach to identify the relevant articles. PubMed, Scopus, and Google Scholar databases were searched for the following keywords: (mental health OR mental illness(es) OR anxiety OR depression OR irritability) AND (elderly OR older people OR aged 60 years or more) AND (Covid-19 OR pandemic OR chronic diseases) AND (mitigate OR manage). In the initial search, we found 948 articles related to our search string, and only 33 studies were included in this narrative review. The results demonstrated that the elderly population is more prone to mental health issues associated with COVID-19. This narrative review also reported that loneliness, stress, depression, anxiety, sleep disturbance, and suicidal ideation symptoms are experienced by the elderly during the pandemic. Our results also demonstrate that interventions, such as community activities through social interactions, and the use of digital technologies could improve the quality of life of older people and help in the mitigation and management of the adverse effects of COVID-19.

## Introduction and background

Coronavirus disease 2019 (COVID-19) is no longer a strange viral infection. Its discovery in late December 2019 at Huanan Seafood Market, Wuhan, China, affects humanity in economic, social, physical, and psychological manner [1]. Since the onset of the COVID-19 outbreak, authors have drawn attention to a variety of mental health issues, including stress, anxiety, and depression [2,3]. A substantial factor in illness is poor mental health and well-being, with depression being the main culprit [4]. Recently, a review on the detrimental psychological impacts of confinement during a quarantine was conducted [5] that includes confusion, post-traumatic stress symptoms, anger, depression, emotional disturbance, low mood, stress, anxiety, insomnia, and irritability. The older population needs special consideration in this situation since they have a greater risk of developing serious illnesses [6]. In this regard, a recent study indicated that adults over the age of 60 were more likely to experience depression and have a bad quality of life due to their health during the continuing pandemic, especially if they exhibited COVID-19 symptoms [3]. Therefore, older people are more likely to get SARS-CoV-2 than younger people and have a worse prognosis.

This narrative review describes the mental health problems related to COVID-19 in the older population and based on current scientific knowledge explains (i) the relation between becoming older and the appearance of greater adverse effects when affected by COVID-19; (ii) the extent to which the daily routine of older people is affected due to COVID-19; (iii) the extent of mental health issues faced by the elderly; (iv) different factors influencing the risk of mental illness in older patients during the pandemic (such as sex, age, location, living conditions, and others); (v) the prevalence of different mental health issues (anxiety, depression, and irritability) in the elderly, especially in people aged 60 years exposed to the negative impact of the COVID-19 pandemic; (vi) the relationships between isolation and mental health issues for elderly people; (vii) the relationship between sociodemographic variables, chronic diseases, and mental health issues for elderly patients; and (viii) the different methods to mitigate and manage the adverse effects of COVID-19 in the elderly.

## Review

Methods

The findings from both quantitative and qualitative investigations were combined in a narrative review that we conducted [7]. Instead of using a systematic review methodology, we chose a narrative review strategy since it allowed us to synthesize and connect research with various methodological and theoretical conceptualizations [8,9]. The research that was relevant to this review featured a variety of study designs, and many of them did not follow conventional methods for gathering data and theoretical precepts. To identify relevant articles (2019-2022), we conducted keyword searches using PubMed, Scopus, and Google Scholar databases. The keywords searched were as follows: (mental health OR mental illness OR anxiety OR depression OR irritability) AND (elderly OR older people OR aged 60 years or more) AND (Covid-19 OR pandemic OR chronic diseases) AND (mitigate OR manage). These terms were meant to assist us find the publications that addressed our research questions. From these keyword searches, we searched and chose peer-reviewed research publications that were written in English. By looking through the references cited in the papers, we discovered through our keyword searches other publications utilizing the snowball search technique. Preprints were excluded since we could not verify their robustness as these papers had not yet been formally acknowledged by the scientific community through the peer-review process.

We disqualified studies during the first title and abstract screening if they (i) dealt with unrelated subjects (e.g., the title or abstract did not match our research objectives), (ii) targeted a younger demographic, or (iii) were not original research. As we did not use a systematic strategy, we kept our choice of studies open and took into account any studies that addressed at least one of our study topics, even though we carefully selected articles from our searches. Instead of documenting every conceivable study that might have been conducted on these themes, our goal was to consolidate the existing literature on mental health problems linked to COVID-19 in older populations.

Results

In our first search, we discovered 948 papers in the title and abstract search from databases of PubMed, Scopus, and Google Scholar that were pertinent to our sting. Due to duplicate data, irrelevant studies, meta-analyses, and systematic review articles, 915 articles were discarded after the screening. This narrative evaluation was limited to 33 papers and separated into pertinent sections.

Relation Between Becoming Older and the Appearance of Greater Adverse Effects

A research by Isaacs et al. (2018) [10] found that elderly adults frequently experience psychological discomfort. Meng et al. (2020) [11] evaluated the psychological health of an older population in China during the COVID-19 pandemic in different research. According to their findings, COVID-19 was causing anxiety and sadness in 37.1% of the aged patients. The emotional reaction is more pronounced in the population over the age of 60, according to Qiu et al. (2020) [12] who reported that emotional response is more prominent in population aged >60 years. These results showed that older age patients are more vulnerable to the negative side effects of COVID-19.

Daily Routine and Mental Health of Older People During COVID-19

According to a research by Armitage and Nellums (2020) [13], older adults experience greater social withdrawal and loneliness during quarantine, which has a serious negative impact on their everyday lives and their mental health. It has been suggested that the anxiety and loneliness of a protracted quarantine might have psychological or mental consequences and problems that result in anxiety and stress [13,14]. According to one study, stress, sadness, anxiety, and a weakened immune system have a detrimental impact on well-being and cause socioeconomic hardship [15]. Santini et al. (2020) [16] also showed that older people who are socially isolated are more likely to experience anxiety and sadness, particularly if they have no close family or friends or are receiving social care or volunteer services.

Factors Influencing the Risk of Mental Illness in Older Patients

The amount of total energy used by senior citizens in Italy for physical exercise each week has significantly decreased, according to Maugeri et al. (2020) [17]. Similar information was discovered by Pérez et al. (2021) [18] in Spain, Meyer et al. (2020) [19] in the United States, Qin et al. (2020) [20] in China, and Yamada et al. (2020) [21] and Yamada et al. (2021) [22] in Japan. These statistics show a decline in physical activity on a worldwide scale, highlighting the necessity of developing methods to improve this situation wherever it occurs. Table 1 shows the results of research illustrating the factors influencing the risk of mental illness depending on age and location.

**Table 1:** Research study characteristics COVID-19: coronavirus disease 2019

Author (year)	Study design	Location/country	Sample size (n)	Age (years)	Findings
Maugeri et al. (2020) [17]	Cross-sectional	Italy	296	60	Elderly people reported having less total energy for physical activities each week, which had an effect on their mental health causing anxiety and depression
Pérez et al. (2021) [18]	Cross-sectional	Spain	98	82.4±6.1	In fragile elderly community dwellers, depressive symptoms, weariness, and social interactions affected physical activity
Meyer et al. (2020) [19]	Cross-sectional	United States	1062	≥65	During COVID-19, the elderly population reported less physical activity
Qin et al. (2020) [20]	Cross-sectional	China	184	≥60	The elderly with a sedentary lifestyle and insufficient exercise
Yamada et al. (2021) [22]	Cohort	Japan	937	73.5	Due to a decline in physical activity during the pandemic, older persons who live alone and are socially inactive are more prone to have incident frailty or impairment
Yamada et al. (2020) [21]	Cross-sectional	Japan	1600	74±5.6	The level of activity dropped in older people
Forlenza and Stella (2020) [23]	Self-assessment in a clinical sample of psychogeriatric patients	Brazil	72	>60	The elderly reported sleep issues such as insomnia, insufficient sleep, or daytime sleepiness. The elderly reported experiencing anxiety, despair, and dysphoria as their mood symptoms
Meng et al. (2020) [11]	Cross-sectional by self-developed questionnaire	China	1556	≥60	Nearly 40% of older people reported having anxiety or depression
De Pue et al. (2021) [24]	Cross-sectional by online survey	Belgium	639	≥65	According to reports, older people's well-being, which is associated with depression, along with their physical activity and sleep quality, all significantly declined throughout the epidemic. During the COVID-19 epidemic, depression in the elderly was linked to a lack of pleasure with life

Prevalence of Different Mental Health Issues

The frequency of mental health problems among elderly persons during COVID-19 varied. According to Mowla et al. (2022) [25], 87% of the survivor group had depression compared to 47% of the control group, and 93% of the survivor group had anxiety. The high rates of psychological issues show how common anxiety and despair were throughout the pandemic, not just among those who survived the illness but even among those who were not affected. Additionally, anxiety (42%), sadness (31%), insomnia (40%), post-traumatic stress disorder (32%), and obsessive-compulsive disorder (20%) were also observed in a study of 402 COVID-19 survivors [26]. Despite having lower inflammatory markers, females were shown to have higher levels of despair and anxiety, which may explain why females typically report depression at a 2:1 ratio to males. At the follow-up examination of this cohort, inflammatory markers were also the predictors of depression and anxiety in addition to a prior psychiatric history, although gender was not. However, Das et al. (2021) [27] found that among the older population (>60 years), anxiety and depression were present at the rates of 8.7% and 15.2%, respectively.

Isolation and Mental Health Issues

According to research by Zaninotto et al. (2022) [28], the prevalence of clinically significant depressive symptoms increased from 12.5%, which was before the COVID-19 pandemic, to 22.6% in June and July 2020 and then increased again to 28.5% in November and December 2020. This was followed by a worsening of life quality and an increase in loneliness. The prevalence of anxiety increased from 9.4% (during June and July 2020) to 10.9% (during November and December 2020). Similar to this, Lábadi et al. (2022) [29] noted that loneliness and catastrophizing had a detrimental impact on changes in mood, social connectivity, and quality of life.

Chronic Diseases and Mental Health Issues

After the start of the COVID-19 outbreak, Wong et al. (2020) [30] found that older patients with multimorbidity in primary care had worse psychosocial health and a rise in missed appointments for chronic disease. Having more chronic diseases, being a female, and living alone were all linked to lower outcomes. According to two studies, persons aged 65 and older and those who have underlying medical illnesses including diabetes, hypertension, or cardiovascular disease are at a higher risk of getting more severe COVID-19 consequences [31,32]. The frequency of stress or other psychological disorders also impacted the immune system [15]. The epidemic and seclusion may also create dementia-related psychological problems in the elderly [33]. The pandemic puts the elderly at risk for well-being issues such as frailty, loneliness/social isolation, serious illness, cognitive impairment, psychological restraint, informational myths, the lack of access to COVID-19 testing, psychosocial vulnerability, addiction disorders, and the difficulties associated with connecting with others online [34]. Age-related anxiety and sadness are more likely to affect seniors who are confined to their homes, socially isolated, and disconnected from society [13].

Mitigation and Management of the Adverse Effects of COVID-19

Older persons who experience loneliness need interventions, such as community activities through social connections, to improve their quality of life. In addition, a randomized controlled trial study in China involving 72 patients with COVID-19 who were 65 years of age or older has demonstrated that a six-week respiratory rehabilitation program can not only enhance the quality of life and respiratory function but also lessen anxiety in elderly COVID-19 patients [35]. During this pandemic, it is crucial to take care of the health and wellness of the elderly (>60 years) [34]. It has been shown in a different cross-sectional study involving 483 people with an average age of 65.49±5.14 that older adults who regularly engaged in vigorous physical activity (VPA) and moderate-vigorous physical activity (MVPA) during the quarantine reported higher scores in resilience (locus, self-efficacy, and optimism), positive affect, and lower depressive symptoms [36].

Similar to how older people can be educated, supported, and counseled using digital technologies for therapeutic treatments and psychological services [23,37,38], social support via internet channels such as WhatsApp, Twitter, and Facebook, as well as online choirs and religious services, may boost interactions between the elderly and other people on a social level [24,39]. Utilizing digital tools such as cellphones or video conversations could help lessen the loneliness brought on by physical separation [40]. According to Tsai et al. (2010) [41], in an evaluation of a video conferencing program designed to foster interaction between elderly people and their families, they discovered that people who used video conferencing experienced less loneliness. It has been discovered that keeping busy and cultivating a sense of friendship are effective ways to combat loneliness in elderly adults [42].

Discussion

**Figure 1:**
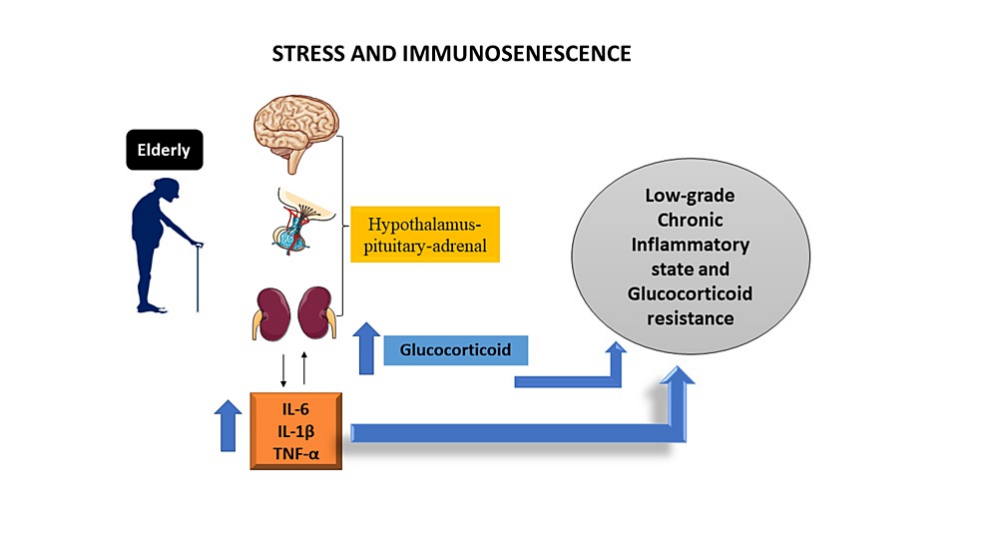
A chronic inflammatory condition results from the immunosenescence that happens with ageing, which is accompanied by a rise in pro-inflammatory cytokines Adapted from Grolli et al. (2021) [45] IL-6, interleukin 6; IL-1β, interleukin 1 beta; TNF-α, tumor necrosis factor-alpha

When compared to younger age groups (<60 years), older (>60 years) patients are more likely to develop severe COVID-19 sickness and are commonly admitted to intensive care units, where fatality rates are higher. Geriatric fragility is primarily brought on by the physical, psychological, and social weaknesses that come with age. This narrative review revealed a significant amount of psychological suffering among older people [10-12]. According to Bektas et al. (2017) [43], the immunosenescence that develops with ageing appears to be linked to a persistent low-grade inflammatory state, which can be a risk factor for aberrant inflammation and increase the severity and mortality risk in aged people [44]. When stress and ageing are taken into account, the hypothalamic-pituitary-adrenal (HPA) axis's relationship to the immune system can set off a vicious cycle that hyperactivates the endocrine and inflammatory systems (Figure 1), making the elderly more prone to both a worsening of COVID-19 symptoms and a worsening of psychiatric disorders.

**Figure 2:**
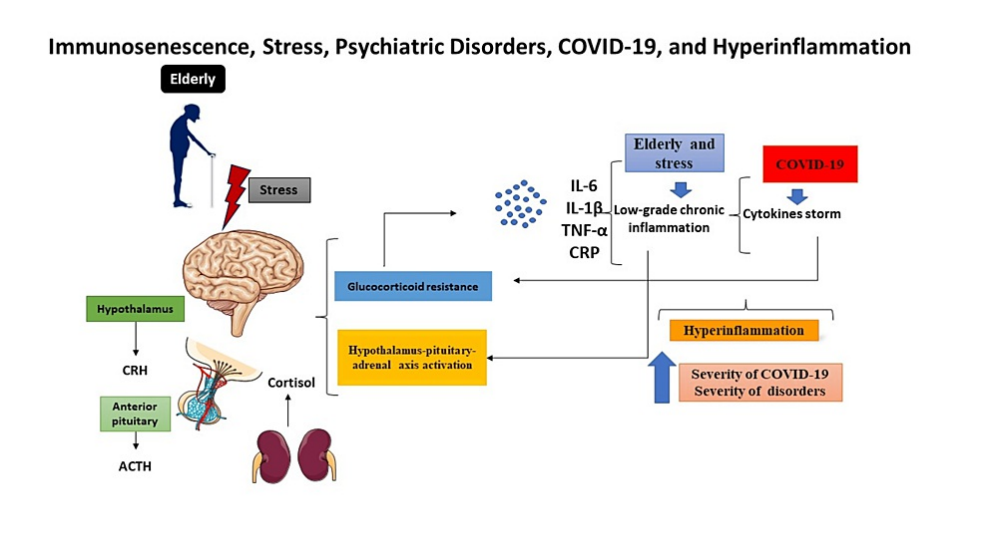
Age-related immunosenescence is characterized by a persistent inflammatory state Adapted from Grolli et al. (2021) [45] CRH, corticotropin-releasing hormone; ACTH, adrenocorticotropic hormone; TNF-α, tumor necrosis factor-alpha; CRP, C-reactive protein; COVID-19, coronavirus disease 2019; IL-6, interleukin 6; IL-1β, interleukin 1 beta

The elderly are reported to exhibit symptoms of suicide ideation, loneliness, stress, sadness, and anxiety throughout the pandemic, according to this narrative review [15,25,26]. In addition, some people may struggle with issues related to brain function, such as the inability to concentrate, remember, recall specifics, or multitask. Seniors' physical and mental health are improved by physical activity such as gardening [18,20]. The synergistic effects of a SARS-CoV-2 infection and a potentially harmful biological condition related to ageing must be carefully considered for potential mental damage. This confluence can lead to a hyper-inflammatory state, a more profound alteration in the HPA axis's function, and the start or worsening of psychiatric diseases (Figure 2).

This review detailed a number of strategies that help elderly people manage or reduce the negative effects of COVID-19 [23,36-38]. Various strategies have been reported in the literature to help older people cope with the pandemic's social isolation, which is likely to last for months. If elderly people are instructed and forced to stay at home, it is crucial to make sure that daily necessities such as groceries and prescription drugs are delivered on time. Additionally, immediate action is required to lessen the negative effects of social isolation on both mental and physical health [13].

## Conclusions

The findings showed that older people are more likely to experience COVID-19-related mental health problems. The elderly are said to endure feelings of loneliness, stress, sadness, anxiety, sleep disturbance, and suicidal ideation during the pandemic, according to this narrative review. Our findings also show that interventions such as social contact in the community and the use of digital technology can enhance older people's quality of life while also assisting in the management and mitigation of COVID-19's negative effects. The elder population reported mental health issues during COVID-19, which might be managed or minimized by implementing social interventions.
